# Brain transcriptomics of agonistic behaviour in the weakly electric fish *Gymnotus omarorum*, a wild teleost model of non-breeding aggression

**DOI:** 10.1038/s41598-020-66494-9

**Published:** 2020-06-11

**Authors:** Guillermo Eastman, Guillermo Valiño, Santiago Radío, Rebecca L. Young, Laura Quintana, Harold H. Zakon, Hans A. Hofmann, José Sotelo-Silveira, Ana Silva

**Affiliations:** 1 Departamento de Genómica, Instituto de Investigaciones Biológicas Clemente Estable, Ministerio de Educación y Cultura, Montevideo, Uruguay; 2 Unidad Bases Neurales de la Conducta, Instituto de Investigaciones Biológicas Clemente Estable, Ministerio de Educación y Cultura, Montevideo, Uruguay; 3 Department of Integrative Biology, The University of Texas, Austin, Texas USA; 4 Center for Computational Biology & Bioinformatics, The University of Texas, Austin, Texas USA; 5 Department of Neuroscience, The University of Texas, Austin, Texas USA; 6 Sección Biología Celular, Facultad de Ciencias, Universidad de la República, Montevideo, Uruguay; 7 Laboratorio de Neurociencias, Facultad de Ciencias, Universidad de la República, Montevideo, Uruguay

**Keywords:** Genome-wide analysis of gene expression, Sequence annotation, Transcriptomics, Social behaviour

## Abstract

Differences in social status are often mediated by agonistic encounters between competitors. Robust literature has examined social status-dependent brain gene expression profiles across vertebrates, yet social status and reproductive state are often confounded. It has therefore been challenging to identify the neuromolecular mechanisms underlying social status independent of reproductive state. Weakly electric fish, *Gymnotus omarorum*, display territorial aggression and social dominance independent of reproductive state. We use wild-derived *G. omarorum* males to conduct a transcriptomic analysis of non-breeding social dominance relationships. After allowing paired rivals to establish a dominance hierarchy, we profiled the transcriptomes of brain sections containing the preoptic area (region involved in regulating aggressive behaviour) in dominant and subordinate individuals. We identified 16 differentially expressed genes (FDR < 0.05) and numerous genes that co-varied with behavioural traits. We also compared our results with previous reports of differential gene expression in other teleost species. Overall, our study establishes *G. omarorum* as a powerful model system for understanding the neuromolecular bases of social status independent of reproductive state.

## Introduction

The search for both the neuromolecular basis of aggression and a conserved transcriptomic signature of dominance across species are matters of current research^1,2^. Social behaviour in vertebrates arises from a network of brain nuclei named the Social Decision-Making Network (SDMN), which includes a highly interconnected group of conserved brain regions^3–6^. It is proposed that the patterns of neural activity occurring among these SDMN nodes may control social behaviour. The plasticity that produces a different weighting of activity across the network can also produce the diversity in social behaviour^4,7^. In addition, it is well known that neuromodulators shape the spatio-temporal pattern of activity of the network in a context-dependent manner^4,5,8–10^. This plasticity of the SDMN by which it adapts its activity to different social demands is driven, at least in part, by modulation of neural gene expression^11–13^. However, when the same circuits of the SDMN are engaged in different social behaviours at the same time, as occurs in male reproductive competition (the stereotypical type of aggression studied so far^14^), identifying the neural gene expression patterns associated with distinct behaviours is difficult to do. Thus, characterizing the neuromolecular basis of social behaviour requires decoupling individual behavioural phenotypes empirically. There are at least two ways to address this confound by comparative studies identifying common transcriptomic signatures of social dominance in males and females^1,15^ or in males inside and outside the breeding season^16^, as we do in this study.

One clear example of behavioural plasticity arises from the establishment and consolidation of the dominant-subordinate status. The strong behavioural asymmetry between contenders during agonistic encounters has provided a solid base for the search of phenotype-dependent gene expression profiles in the brain of vertebrates^17^. In particular, in fish, multiple reports have shown an association between social status and the variation of a few candidate genes^6,18,19^, as well as changes in gene expression at a genome-wide scale^1,2,6,20–28^. A common result of measuring dynamic responses in gene expression is that there are hundreds to thousands of genes that are differentially expressed as a function of dominance, many of which have unrelated functions^28,29^. Another generality is that these studies are often limited by their use of complex tissue, such as the whole-brain, which prevents drawing conclusions about gene expression in specific brain regions that are important for social behaviour^30^. In addition, two more issues complicate the interpretation of genome-wide data in this type of experiments. First, the physiological changes associated with dominance occur in a reproductive context and dominance hierarchies are often mediated through hormones that are already high and may already be affecting gene expression. Careful experimental designs are therefore necessary to distinguish gene expression associated with status rather than with reproductive physiology^6,31–33^. Second, differences in resting gene expression between behavioural types, such as long-term established social hierarchies are likely to reflect processes that are involved in maintaining rather than triggering a particular neurogenomic state^34,35^.

To avoid the bias reproductive physiology might have on the neuroendocrine mechanisms of social dominance, we worked with the South American weakly electric fish *Gymnotus omarorum*, a well-established model system of non-breeding aggression among teleosts^36–41^. Since the discovery of active electroreception^42^, weakly electric fish have become a popular model system in diverse disciplines that include neuroanatomy, endocrinology, biophysics, ecology, and evolution (reviewed in Pitchers *et al*. 2016). More recently, next-generation sequencing and modern molecular genetic techniques have had a profound impact on this field^43–47^. However, genome-scale resources are currently available for only a few species of weakly electric fish, and there is only one pulse-type gymnotiform species, *Brachyhypopomus gauderio*, in which genomic engineering techniques have been undertaken so far^48^.

*Gymnotus omarorum* is a sexually monomorphic species that occurs at the southernmost boundary of gymnotiform distribution in South America^49^. This species is a seasonal breeder, with reproduction restricted to three months of the year but with territorial aggression displayed year-round. Both males and females hold same-sized territories in the wild across seasons. Interestingly, this territorial aggression is strikingly robust during the non-breeding season, and is mediated by a well characterized agonistic behaviour^37,38^. Once a social hierarchy is established, there is no reversion of contest outcome for at least 2 days, and dominants remain highly aggressive against subordinates, which only signal submission in a precise sequence of locomotor and electric displays^37,38,40^. The non-breeding territorial aggression of *G. omarorum* thus occurs uncoupled from the reproductive state, when gonads are quiescent. The resulting dominance is uncorrelated to levels of circulating steroid sexual hormones, and this aggression remains unchanged in castrated animals^36,37^. Gonadal independent mechanisms of the control of non-breeding aggression are being actively studied in the brains of wild species of mammals and birds^50,51^, and it is hence timely to add a corresponding teleost model system to this perspective.

To disentangle the neuromolecular mechanisms driven by reproduction or by dominance, we performed a genome-wide study using a wild model of non-breeding territorial aggression in the weakly electric fish *Gymnotus omarorum*. Specifically, we examined the transcriptomes of the preoptic area in socially dominant and subordinate males 36 hours after they initiated aggressive displays, which is a valuable time window to evaluate transcriptomic changes involved in the establishment of social hierarchies. Our results make several complementary contributions; first, we present the reference transcriptome of *G. omarorum*, a useful resource for further studies in this species. Second, we communicate a genome-wide study linking brain gene expression with dominant-subordinate status and agonistic behaviour. Last, we compare our transcriptomic dataset with other previously described datasets, looking for conserved expression patterns of dominance in teleosts. This study thus contributes a novel non-breeding teleost model of dominance to shed light into neuromolecular bases of dominance across vertebrates.

## Results

### Transcriptome assembly and annotation

**Figure 1:**
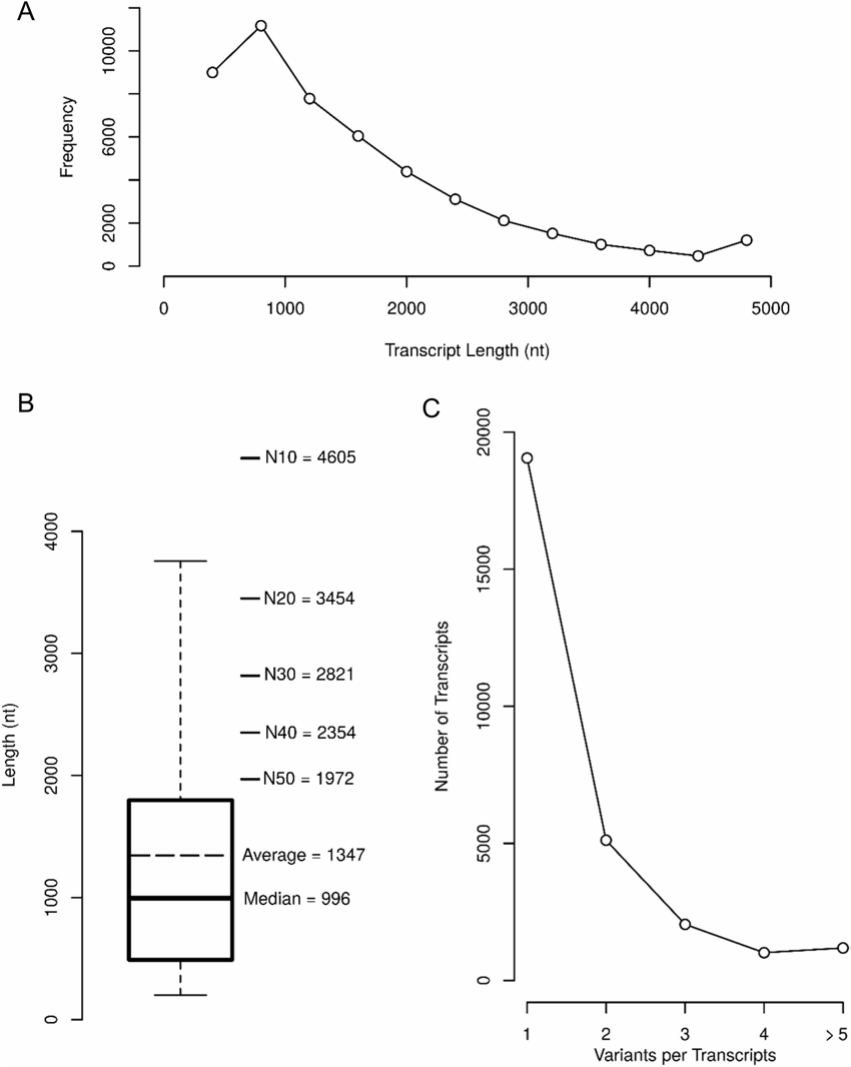
Assembled transcriptome reference description. (**A**) Transcript length distribution is shown from 200 nt (shortest assembled transcript) to 5000 nt (longest assembled transcripts is 17,998 nt in length). (**B**) Same as (A) but as a boxplot distribution indicating median, average and N50 values, among others. (**C**) Total number of transcripts with different transcripts variant is shown.

Due to the absence of genomic resources for gymnotiform species we first generated a reference transcriptome for *G. omarorum*. For this, we isolated RNA from different tissues (brain, electric organ, muscle and spinal cord) obtained from two individuals. Following the pipeline described in Supplementary Fig. S1, a reference transcriptome was assembled and annotated. A total of 48,522 different transcripts variants were identified, which represent 28,417 transcripts in the annotated transcriptome. Almost half of annotated transcripts were longer than 1Kb (24,211), while 51 were longer than 10Kb, showing an expected size distribution (Fig. 1A). Several statistical parameters describing transcripts sizes are shown in Fig. 1B. For example, transcript size median and average was 996 and 1,347 bases respectively, the N50 obtained was 1,972 and GC content was 48.33%, among others quality parameters, indicating a high quality *de novo* assembly (see similar results in Gallant et al. 2014 and Traeger *et al*. 2015). The number of transcripts variants per transcript is shown in Fig. 1C, with almost 20,000 transcripts with only one variant. The assembled and annotated transcriptome is available at www.efishgenomics.integrativebiology.msu.edu.

### The establishment and consolidation of the dominant-subordinate status

**Figure 2:**
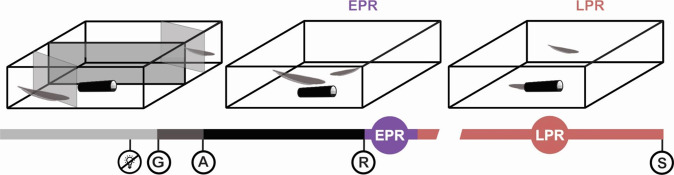
Schematic representation of the behavioural paradigm. Two males were placed in opposite sides of a 120-L tank with two extra partitions located on opposite corners preventing any physical or electrical interaction before gate removal (left panel). Lights were turned off at dawn (lightbulb icon) and 5 min later all gates were removed (G) to let the males interact. The first attack (**A**) initiates the conflict phase, and resolution (R) is achieved when the subordinate retreats 3 times without attacking back. Post-resolution was recorded for 36 h before removing and sacrificing (S) both the dominant and the subordinate fish for RNA-Seq. The post resolution period was divided into two phases: the first hour was considered an early post resolution phase (EPR, middle panel), and the remaining 35 hours were considered as the late post resolution phase (LPR, right panel). In both phases several behavioural parameters were measured and evaluated. Image modified from Perrone *et al*. 2019.

**Figure 3:**
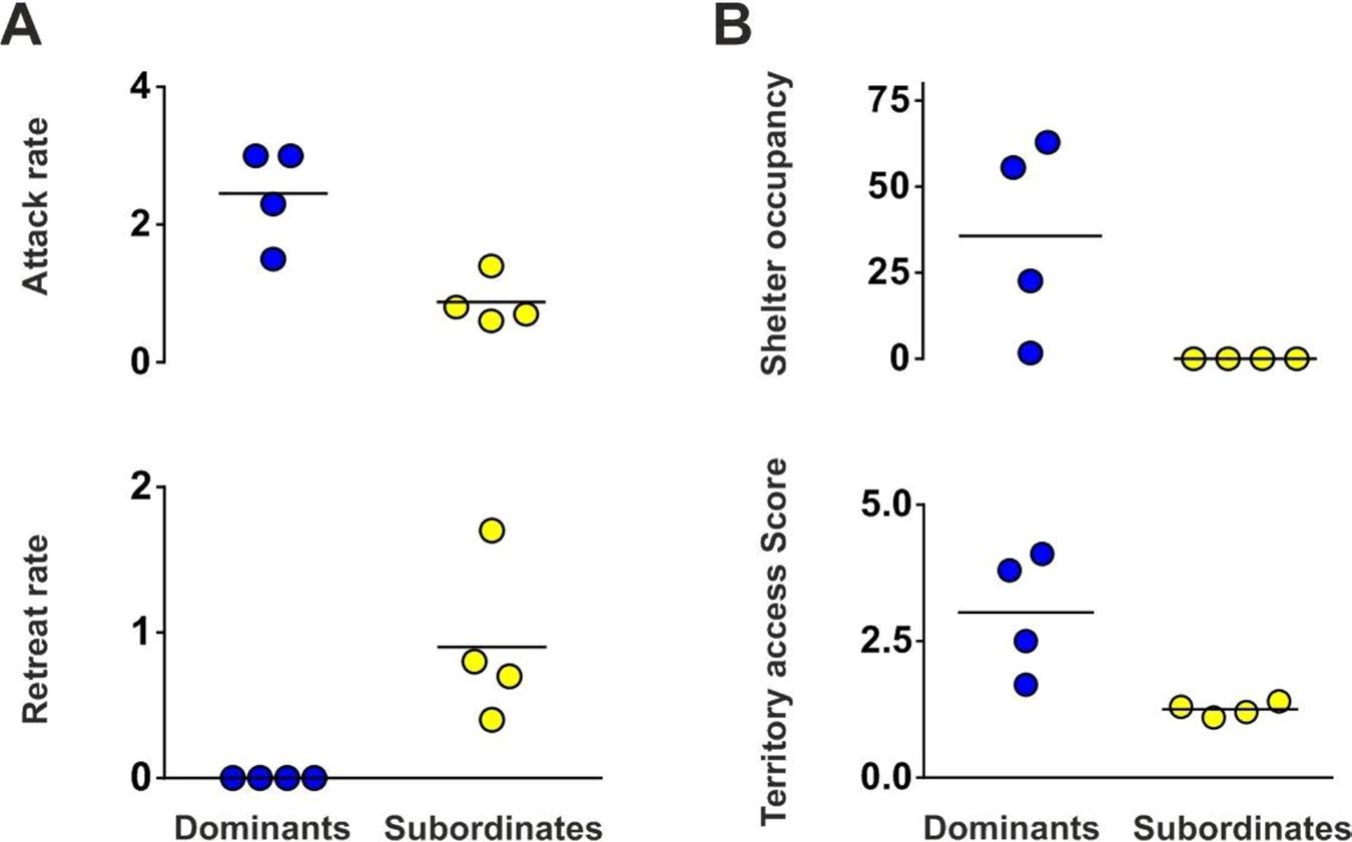
Asymmetries in the behaviour of dominants and subordinates during (**A**) the contest and (**B**) late post resolution phase (LPR). Attack and retreat rates were evaluated in the context phase (n/min) while shelter occupancy (%) and territory access score were determined in the LPR phase. Horizontal lines represent mean value (n = 4).

The territorial behaviour of *G. omarorum* is mediated by agonistic encounters all year round. Its exploration during the non-breeding season provides an unparalleled opportunity to identify the neuromolecular substrates of social dominance independent of reproductive status. The non-breeding agonistic behaviour of *G. omarorum* was tested in male-male dyadic encounters in a plain arena in which space is the only resource animals compete over (Fig. 2). As previously reported^39^, all dyads (n = 4) represented unambiguous instances of the emergence and consolidation of the dominant-subordinate status over the course of the interaction (36 h). All encounters included a short evaluation phase (39.3 ± 28.0 sec, M ± SD) and were resolved in a maximum of 8 min (267.3 ± 156.8 sec, M ± SD). The larger male always won the fight and the contest outcome was maintained without reversion during the entire recording period. Dominants and subordinates displayed very different behaviours not only during the contest but also during the post-resolution phase (Fig. 3). During contest and post-resolution, dominants attacked but never retreated, while subordinates retreated in all phases but only attacked during contest. Subordinates also occasionally emitted electric signals of submission (offs and chirps^38^) during the early post-resolution (EPR). In addition, during the late post-resolution phase (LPR), dominants (but not subordinates) clearly held the conquered resource by exhibiting an exclusive access to the shelter and a privileged use of its surroundings (Fig. 3).

### Transcriptomic analysis

**Figure 4:**
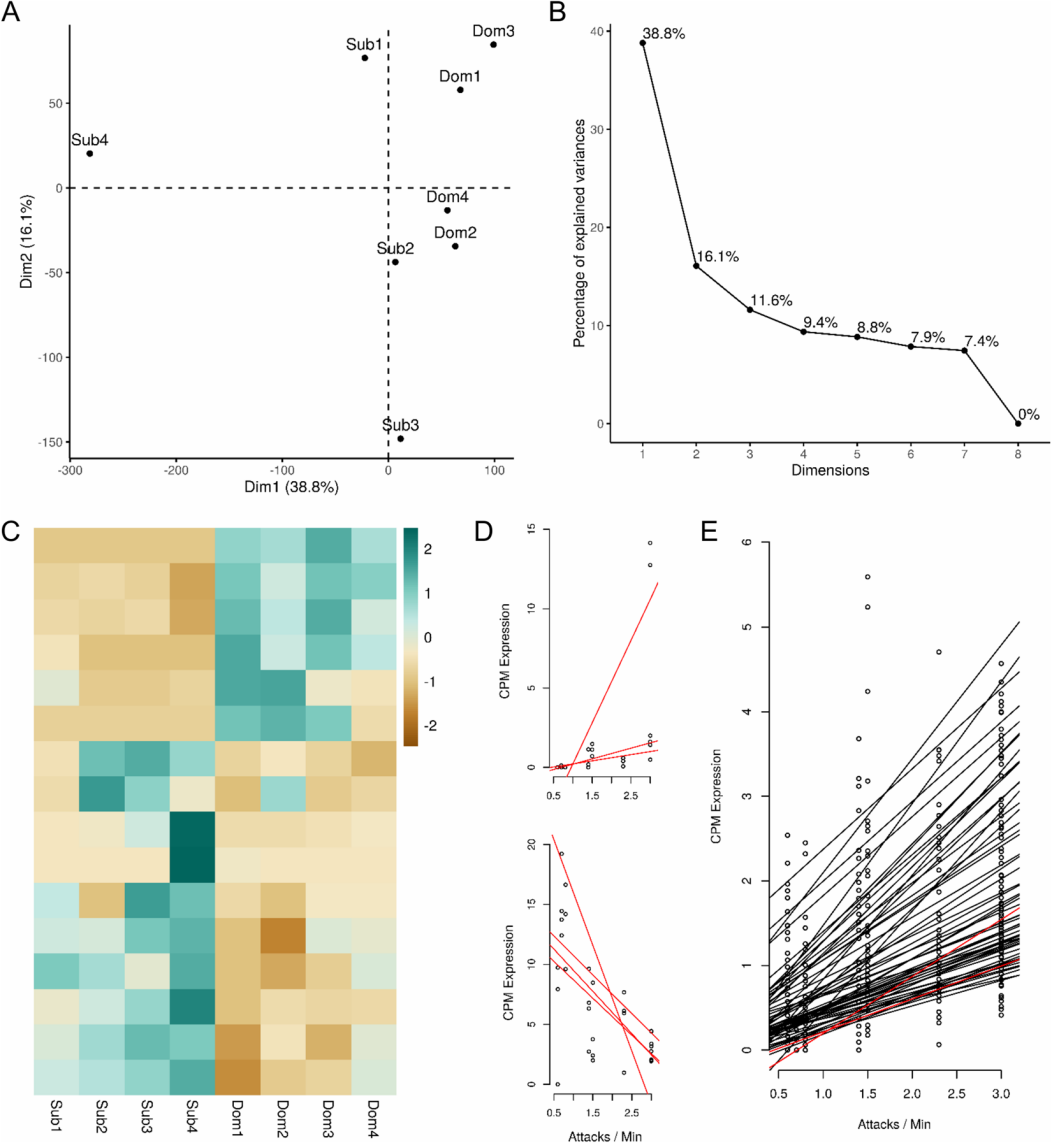
Behaviour asymmetries are reflected in transcriptomic data. (**A**) Principal Component Analysis of RNA-Seq samples separates subordinates from dominants by dimension 1. (**B**) Scree plot showing percentage of explained variation by each dimension of the PCA. (**C**) Heatmap showing expression levels of the 16 DEGs (FDR < 0.05). (**D**) CPM expression levels are plotted vs the attack rate for 7 out of the 16 DEGs that present a high and significant Pearson correlation (ρ > 0.7 and associated p value <0.05). (**E**) CPM expression levels are plotted against the attack rate for a subset of 60 genes that load most strongly on PC1 and present high and significant Pearson correlation (see above). In (D) and (E) red lines represent genes with FDR < 0.05.

RNA-Seq data obtained from brain sections containing the preoptic area (POA) of dominants and subordinates (see Methods) consisted of 10–16 million paired-end reads per sample. After quality trimming, reads were mapped against the annotated *G. omarorum* transcriptome described above (see Methods), obtaining almost 50% of mapping in each case (Supplementary Table S1). We were able to map expression signals to 96% of the reference, and almost 15,000 transcripts had an average of 10 or more reads in both conditions. Principal component analysis (PCA) showed a clear separation between dominants and subordinates by PCA component 1, which explains almost 40% of variance (Fig. 4A,B), and also anticipates that subordinates are more variable than dominants (Supplementary Fig. S2A). We then analysed transcriptome-wide patterns of expression levels as well as inter-replicate correlations (Supplementary Fig. S2B-E). Next, we identified 16 differentially expressed genes (DEGs) between dominants and subordinates, using the standard threshold >2-fold difference in expression and an associated false discovery rate (FDR) less than 0.05 (Fig. 4C and Supplementary Table S2). The expression levels of these 16 DEGs were correlated with the behavioural measures. Interestingly, expression levels of 10 of these 16 genes (62.5%) correlated with at least one behavioural trait. Specifically, seven genes (43.8%) show a high and significant correlation (Pearson correlation value ρ > 0.7 and an associated p-value < 0.05) with the attack rate (Fig. 4D), including somatostatin (fold-difference = −7.51 and FDR = 1.63.10^−03^), a peptide hormone involved in the regulation of somatic growth and previously reported in another teleost to regulate aggression in a dose-dependent manner^52^. In addition to the strong association between the small number of DEGs we detected and behavioural traits, we also found that 266 of the 500 genes (53.2%) that most strongly loaded on PC1 were strongly correlated (|ρ | > 0.7, p-value < 0.05) with behavioural traits. Sixty of these genes (12.0%) were strongly correlated with attack rate at ρ > 0.7 and p-value < 0.05 (Fig. 4E).

### Searching for a conserved gene expression signature of social dominance

**Figure 5:**
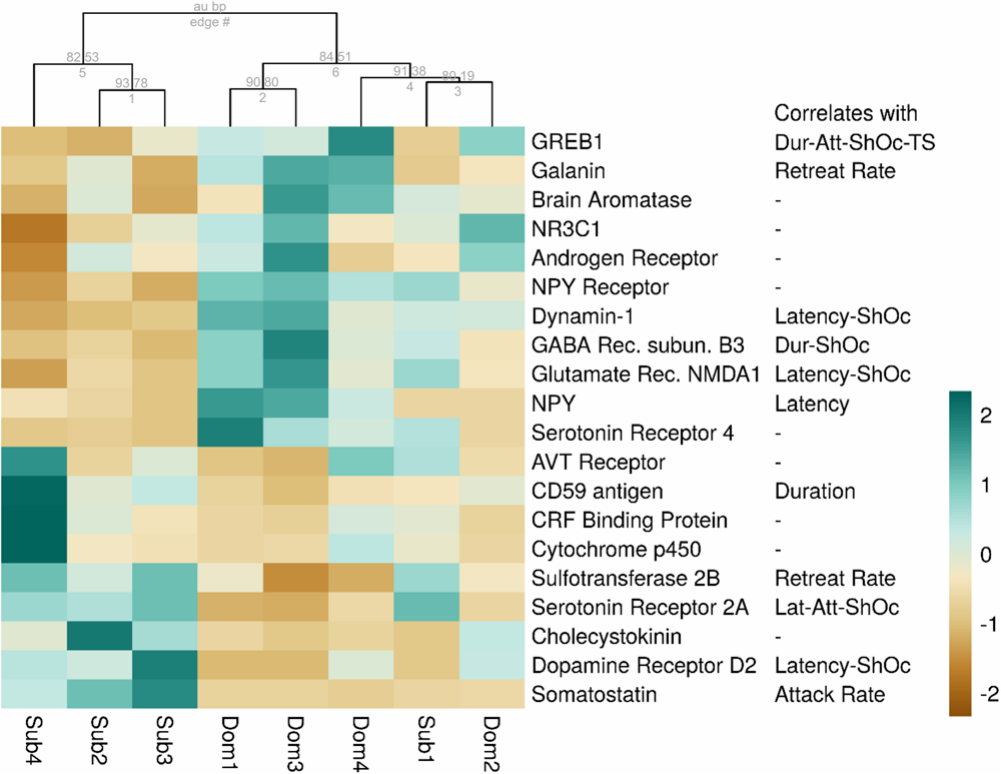
Heatmap showing expression of genes associated with social dominance obtained from the literature and/or related to our own behavioural/pharmacological work. Behaviour traits that correlate (|ρ | >0.7 and p value <0.05) with gene expression are indicated in each case. Bootstrap support values are shown.

With the aim of identifying a dominance associated transcriptomic signature, we explored previous studies of status-dependent gene expression among teleosts to build the heatmap presented in Fig. 5 using the following criteria. First, we used a text-mining tool (developed in-house, manuscript in preparation; https://github.com/sradiouy/IdMiner) to mine PubMed abstract database to capture previously reported associations of social behavioural terms within the list of 16 DEGs (Supplementary Table S2). We retrieved the gene for somatostatin, previously described here and elsewhere as a regulator of aggressive behaviour^52^. Second, we selected transcripts orthologous to genes previously described as more highly expressed in dominant (neuropeptide Y -NPY-, androgen receptor variants and glucocorticoid receptor -NR3C1-) and subordinate zebrafish (dopamine receptor and corticotropin releasing factor -CRF-), respectively^25,53,54^ (Supplementary Table S3). Third, a similar strategy was used to match our data with the candidate genes associated with social dominance status in the African cichlid fish *Astatotilapia burtoni* (reviewed in Maruska & Fernald, 2013). In the latter, the following genes present a conserved expression pattern: CD59-like protein, GABA receptor β subunit, dynamin-1, aromatase, androgen receptor, galanin, and cholecystokinin. Finally, we selected relevant dominant and subordinate overexpressed transcripts based on our previous behaviour and pharmacological work^36,40,41^. For example, sex steroid hormones are known key regulators of aggressive behaviour. We found that in *G. omarorum*, the POA of dominants and subordinates varies in the expression of genes that encode enzymes related to the androgenic and estrogenic pathways, particularly in those of androgen and oestrogen production (Supplementary Fig. S3). Specifically, dominants had a higher expression of aromatase transcripts, the enzyme that mediates the conversion of testosterone into oestrogen, and androstenedione to oestrone. Subordinates, on the other hand, showed a differential increase in transcripts of DHEA sulfotransferase mediating the conversion of DHEA to DHEA sulphate and of Cyp450 1B1 (EC 1.14.14.1), which mediates the conversion from DHEA to 16a-hydroxyl-DHEA and estrone/estradiol into estriol and 2-Hydroxyestradiol. Other genes identified by these criteria included GREB1^55^, AVT receptors^56,57^, serotonin receptors^41,58^, and glutamate receptors^59^. Figure 5 shows that the expression values we measured in *G. omarorum* for this gene set robustly clustered according to social phenotype. When we explored the correlation between the expression levels of these genes with the behavioural traits we observed a significant association in 11 genes (Fig. 5). This group of transcripts represents a putative gene expression signature widely associated with dominant-subordinate status across teleosts.

## Discussion

In this study, we took advantage of the establishment of dominance and subordination that is displayed uncoupled from reproduction in the weakly electric fish, *Gymnotus omarorum* to explore patterns of differential gene expression associated with the dominant-subordinate status in an important area of the SDMN. This is not only the first genome-wide study to link gene expression with social behaviour in electric fish, but also the first study to identify candidate genes of dominance in a vertebrate model of territorial aggression unbiased by the reproductive state.

The enormous diversity in experimental approaches and social systems have made it difficult to identify candidate genes associated with social dominance across species^28^. Gene lists produced by transcriptomic analysis are difficult both to interpret and to extrapolate without additional controls to tease apart gene expression associated with other confounding factors. Previous reports have shown, for example, that neurogenomic mechanisms contributing to behavioural plasticity are different from those responsible for static differences among phenotypes^35,60^. Therefore, the genes identified as differentially expressed between teleost phenotypes (dominant-subordinate) or among morphs displaying different levels of aggression may be related to the maintenance of these phenotypes rather than to the agonistic behaviour that mediated the emergence of the social status. Similarly, the genes related to social dominance, which have been exclusively explored during the breeding season in teleosts so far, may be masked by the enhanced expression of genes related to the maintenance of reproductive behaviour. To facilitate the identification of genes related to the dominant-subordinate status outside of a reproductive context, the acquisition of this hierarchy during the non-breeding season of *G. omarorum* emerges as a very advantageous model system for the following reasons: a) it occurs independently of gonadal steroid hormones^36,37^; b) it allows the analysis of the mechanisms of social behaviours in wild-caught animals that reflect the individual variation present in nature; c) in dyadic encounters the conflict is solved within minutes and this contest outcome persists without reversion^39^; and d) while previous to the contest contenders have the same experience and behave similarly, a robust asymmetry in the behaviour between dominants and subordinates emerges after conflict resolution and remains throughout the recording period (Fig. 3).

We present for the first time a reference transcriptome of the weakly electric fish *Gymnotus omarorum*, an important advance as this species has become a powerful model system in integrative neuroscience. We used this reference to perform differential gene expression analysis in RNA from brain sections at the level of the POA contrasting the behaviour of dominants and subordinates. The POA was a natural node to explore, given the extensive reports on its role for neuro-hormonal integration of social behaviours across teleosts (for example Tripp et al. 2017, Maruska & Fernald, 2013 Greenwood et al. 2008). We thus used brain transverse sections in which the POA was entirely included (although obviously we cannot rule out the involvement of other brain areas of the section). Differential expression analysis reveals only 16 genes significantly modulated at FDR < 0.05 (Supplementary Table S2). Although inter-replicate correlation indicates that the dataset is robust (Supplementary Fig. S2B), the low number of DEGs detected is probably due to the relatively small sample size (n = 4) of animals analysed. It is worth considering other reasons that may increase the noise in our experimental set up, like the dispersion of the behavioural response obtained in wild animals in which genetic background can be quite diverse. Nonetheless, solid patterns of transcriptome variation were found between the two behavioural types, even though we cannot be completely confident at the level of individual genes, due to the low power argument described above. Therefore, we were careful in reporting statistically sound results and present exploratory analysis of pattern expression (see below), focusing specifically on a small subset of genes previously associated with aggression in other teleosts models.

One of these patterns is evidenced by PCA and the variance study shown in Fig. 4A-B and Supplementary Figure S2A, respectively. While dominants present a clear and defined response in terms of transcripts expression levels, subordinates showed a diffuse pattern and a broader response. This asymmetry, previously observed in other behavioural transcriptomics studies^6^, suggests that subordinance might not have one clear pattern of gene expression but rather represents the absence of dominants’ pattern. This hypothesis is supported by the fact that subordinates samples show higher levels of variance than dominants, and that inter-replicate correlation, although always high, is lower in subordinates than in dominants (Supplementary Figure S2A,E).

We found that most of the DEGs described here as well as most of the genes that loaded heavily on PC1 (which separated dominants and subordinates individuals) were strongly correlated with behavioural traits (such as intensity of aggression and privileged access to territory) (Fig. 4D-E). Even though these results suggest that POA gene expression and non-breeding territorial aggression in *G. omarorum* are indeed functionally related, they need to be interpreted with caution. As discussed above, future studies will require a greater number of biological replicates to ensure the statistical power needed to discover more DEGs and to solidify the relationship between gene expression profiles and behaviour.

To identify a conserved gene signature of dominance, we contrasted previously identified candidate genes associated with dominance in teleosts with our dataset (Fig. 5). Among these genes we found the gene for somatostatin, a hormone involved in growth, present in our list of DEGs (Supplementary Table S2). Our results fall in line with a previous report in teleosts, in which somatostatin not only inhibits aggressive behaviour independently of any effect on gonadal androgens, but does so in a dose-dependent manner^52^. We also found the genes NPY, galanin and cholecystokinin that have been shown to be involved in food intake mechanisms. Orexigenic protein transcripts were up-regulated in dominant *G. omarorum* (NPY, as in; Filby et al. 2010 galanin as in Renn et al. 2008 and Tripp et al. 2018), while the anorexigenic protein transcript was overexpressed in subordinates (cholecystokinin, as in Renn et al. 2008). Interestingly foraging and social behaviour have been proposed to be interconnected at a mechanistic level, and neuropeptides involved in food intake have been shown to be expressed in key nodes of the SDMN, including the POA, across vertebrates^61^. Dominant *G. omarorum* may be motivated to acquire their territory as a foraging patch. A complementary explanation is that these neuropeptides are involved in maintaining the social status as it had been proposed for other social behaviours^61^. Genes involved in steroidogenic pathways, as aromatase and androgen receptors^18,53,62,63^, were also found (see below). Other genes are involved in multiple processes: CRF binding protein, CD59-like protein, GABA receptor β subunit, dopamine receptor D2 and dynamin-1 were all overexpressed in subordinates as reported elsewhere^1,20,53,64,65^. In sum, these 20 genes represent a transcriptomic signature of dominance among teleosts. Eleven of these genes also showed high and significant correlation (|ρ | > 0.7 and p value < 0.05) with at least one of the behavioural traits we measured (Fig. 5 and Supplementary Table S4).

Previously identified candidate genes were also compared to our dataset to evaluate divergences. Interestingly, three genes associated with dominance in zebrafish were overexpressed in subordinate *G. omarorum* (tryptophan hydroxylase (TPH1b), nitric oxide synthase 1 (NOS1) and histidine decarboxylase (HDC). Therefore, these genes seem to be consistently related to the establishment of hierarchy, regardless of status. In addition, there are several genes overexpressed in dominant zebrafish, which were not overexpressed in our model. This difference is also interesting because it highlights genes that might only be related to reproductive aggression. We also identified a set of significantly DEGs (FDR < 0.05) previously related to behaviour but not directly associated with the dominant-subordinate status such as Neuralized 2^66^, Relaxin Receptor 2^67,68^ and Hemoglobin subunit beta 2^69–71^ (Supplementary Table S2) that need to be further explored in future studies in other model systems.

We were especially interested in analysing a group of relevant genes that were either related to the modulation of the non-breeding territorial aggression of *G. omarorum* or suggested to be candidate genes of dominance in previous studies (Fig. 5). We first focused on the hormonal signatures of dominance and submission, which are hypothesized to enable animals to adapt to the consequences and demands of winning or losing, integrating information for future interactions^72^. Although the non-breeding territorial aggression of *G. omarorum* is independent of circulating gonadal hormones, oestrogens have been shown to play an important role in its modulation^36,37,73^. Neurosteroids, which may be derived from circulating precursors, likely play a critical role in the regulation of non-breeding aggression in mammals and birds (revised in Demas *et al*. 2007). In this study, we show that brain steroidogenic pathways are activated in a status-dependent manner. Dominant males favour the pathway that drives steroids towards oestrogen synthesis (Supplementary Fig. S3), showing an increase in brain aromatase transcripts and GREB1, an oestrogen responsive gene that has been involved in behaviour^55^, as well as an overexpression of androgen receptors. Conversely, subordinate males show increased expression of transcripts involved in the conversion of active androgens into non-aromatisable androgens (Supplementary Fig S3) favouring the conversion of DHEA into DHEA-sulphate by DHEA-sulfotransferase and into 16a-hydroxyl-DHEA by Cyp450 1B1. In teleosts, aromatase has been consistently reported in association to dominance^18,20^ as androgen receptors^18,53,62,74^. However, to our knowledge this is the first study to suggest a status-dependent neurosynthesis of steroid hormones during the non-breeding period in teleosts, as previously reported in other classes of vertebrates^50,75–77^.

We also focused on two well-understood modulators of agonistic behaviour in vertebrates with reported effects in *G. omarorum*. The hypothalamic neuropeptide arginine-vasotocin (AVT) and its mammalian homolog, arginine-vasopressin (AVP) are key modulators of social behaviour in different contexts^56,57^, and is known to modulate the agonistic behaviour of *G. omarorum* differently in dominants and subordinates^40^. Concordantly, in this study, we found that subordinate males show differential expression of AVT receptor in comparison to dominants. Serotonin (5-HT) is known to inhibit aggression in vertebrates^3^ and show a different tone between dominants and subordinates in the teleost brain^41,58^. Serotonin is also involved in both dominants and subordinates during the agonistic encounter of *G. omarorum*^41^. We found three 5-HT receptors subtypes overexpressed in dominants (5-HT4, 5HT2B and 5-HT7) and one in subordinates (5-HT2A) suggesting that 5-HT affects both phenotypes through different cascades^78^.

The differential pattern of brain gene expression between dominants and subordinates of *G. omarorum*, which show robust, gonadal-independent aggression, allowed us to identify a number of candidate genes, whose expression pattern is conserved among teleosts, and that may represent a neural transcriptomic signature of dominance. Among them, we found several enzymes of the brain steroidogenic pathway, reinforcing the role of neurosteroids in the establishment and consolidation of dominance; as well as hormonal, amine, and neuropeptide receptors whose status-dependent actions have been extensively reported. Finally, there are several genes associated with breeding aggression which do not show modulation in our non-breeding dataset and may represent the seasonal imprint on dominance. Overall, the non-breeding territorial aggression of *G. omarorum* constitutes a relevant model system for understanding the genomic basis of hierarchy.

## Methods

### Transcriptome assembly and annotation

To generate a reference transcriptome four samples derived for two different fishes were obtained (for details see *Animals* section below). Samples from brain (fish 1, female), electric organ (fish 1), muscle (fish 1) and spinal cord (fish 2, male) were sliced and sent on RNAlater to the University of Texas. RNA was isolated using the manufacturer instructions for the Maxwell 16 LEV RNA Isolation Kit (Promega, Madison, WI). RNA libraries were prepared and sequenced (paired-end 150 bp; 25 million reads) by the University of Texas Genome Sequencing and Analysis Facility using the Illumina HiSeq platform. After quality control check, libraries were quality trimmed using *sickle* (https://github.com/najoshi/sickle) and pooled. An *in silico* read normalization was applied and *Trinity* software^79^ was used to produce a primary assembled transcriptome. *Bowtie2*^80^ was used to map all libraries against the primary assembly. This primary assembly was evaluated by *transrate*^81^ who defines a subset of “good contigs” from which several software packages were applied to detect ORFs and protein-coding genes: *TransDecoder.LongORFs* (https://github.com/TransDecoder), *blastx*^82^ against all organism and against only to *Danio rerio* proteins, *Trinotate*^83^ and *findorf* (https://github.com/vsbuffalo/findorf). From proteins identified by *findorf*, other software like *InterProScan*^84^ and *ghostKOALA*^85^ were applied to full annotate protein-coding transcripts. Home-made python and bash scripts were used to produce final annotated transcriptome files: nucleotide and amino acids fasta files and gff annotation file (Supplementary Fig. S1).

### Animals

A total of 10 non-breeding wild-caught adult *Gymnotus omarorum*^49^ were used: 1 male and 1 female for transcriptome assembly and annotation as described above, and 8 males for behaviour and differential gene expression analysis. Animals were collected from the field and housed in institutional facilities for 10 to 30 days before the behavioural experiments (detailed methods in supplementary information).

Electric fish collection for experimental purposes was authorized by DINARA (National Direction of Aquatic Resources) and MGAP (Ministry of Agriculture and Fisheries), resolution No. 065/2004. All experimental procedures complied with ASAP/ABS Guidelines for the Use of Animals in Research and were approved by our institutional ethical committee (Comisión Bioética, Instituto Clemente Estable, MEC, 007/05/2012).

### Behaviour

We performed 4 dyadic (male-male) agonistic encounters during the non-breeding season (occurring during the Austral fall-winter) to avoid any other type of agonistic interactions related to reproduction. Two males with body weight asymmetries between 13.2–24.2% were placed in the experimental behavioural tank, which was a plain arena divided by gates into equal compartments, with a single shelter in the middle (Fig. 1). This setup allowed simultaneous electric and infrared-sensitive video recordings. To avoid weight biases we formed dyads in ranges which would ensure dominant/subordinate overlapping (dominants -D-: D1 = 42.2 g; D2 = 32.4 g; D3 = 20.5 g; D4 = 26.5 g, subordinates -S-: S1 = 32.0 g; S2 = 21.8 g; S3 = 17.8 g; S4 = 20.5 g; Supplementary Fig. S4). During the experiment, video and electric recordings were carried out for 35 hours. Agonistic behaviour started approximately 5 minutes after gate removal; the contest phase started with the first attack and resolution was achieved when one of the contenders (the subordinate) retreated 3 times without attacking back. After 36 hours, we removed and sacrificed both dominant and subordinate individuals, for transcriptomic studies.

We analysed the locomotor displays of the tested individuals to identify the 3 phases of the agonistic encounter: a) evaluation phase (pre-contest), from time 0 (gate removal) to the occurrence of the first attack; b) contest phase, from the first attack to conflict resolution (resolution time); and c) post-resolution phase (post-contest), subdivided into 2 phases: the first hour was considered an early post resolution phase (EPR), and the remaining 35 hours were considered as the late post resolution phase (LPR).

To reflect the use of the shelter and patrolling of territory we used: 1) the shelter occupancy and 2) a territory access score (which contemplate the position of each contender relative to the shelter), in order to corroborate that the dominant-subordinate status was maintained over time without reversion (Fig. 2). Additional details are presented in supplementary information.

### Sample preparation and sequencing

After behavioural experiments animals were sacrificed by overdose of 2-phenoxy-ethanol (3.75 µg/mL; Sigma P-1126). Immediately after, brains were dissected on ice and transverse cryo-sections (50 µm) were obtained in rostro-caudal direction until POA was identified following the anatomical landmarks and coordinates established for this species^86^ and for other gymnotiform species^87^. Four 250 µm sections were isolated (11.9 ± 0.9 mg). These sections not only contained the entire POA but also portions of two other nodes of the SDMN: a part of the anterior hypothalamus and a small portion of the node homologous to the extended medial amygdala^86^, among others (see extended Methods in supplementary information). Sections were stored in RNAlater and sent to the University of Texas for RNA isolation and sequencing as described above (Illumina Paired-end 150 bp). Sequence data is available at SRA (PRJNA562683).

### Sequence read processing, alignment, normalization and comparative analysis

After checking sequences quality using *FastQC* software^88^, libraries were quality trimmed by *sickle*. Alignment was performed by *bowtie2* against annotated transcriptome. Transcripts count tables were obtained using *featureCounts* software from *Subread*^89^. By *edgeR*^90^ samples were normalized and evaluated for differential expression. Differential expression was considered if |fold difference | >2 and p-value, adjusted by FDR, were less than 0.05.

### Meta-analysis

When contrasting available data sets with our results, meta-analysis was performed as follows: candidates genes described in literature were downloaded in amino acid fasta format from NCBI web site. Orthologous transcripts were characterized using reciprocal best hit BLAST, with candidate transcripts as query and our *G. omarorum* reference transcriptome as subject. In each case, orthologous transcripts were ordered by bitscore and analysed considering matching in annotation and the best coverage.

## Supplementary information

Supplementary Information.
