# Supplementary material for: Brain transcriptomics of agonistic behaviour in the weakly electric fish Gymnotus omarorum, a wild teleost model of non-breeding aggression
Source: Sci Rep. 2020 Jun 11;10:9496. doi: 10.1038/s41598-020-66494-9 (PMC7289790; doi:10.1038/s41598-020-66494-9)
Supplement: Supplementary file 1 — Supplementary Information. [file 41598_2020_66494_MOESM1_ESM.docx]

**SUPPLEMENTARY INFORMATION**

***Brain transcriptomics of agonistic behaviour in the weakly electric fish* Gymnotus omarorum*, a wild teleost model of non-breeding aggression***

Guillermo Eastman, Guillermo Valiño, Santiago Radío, Rebecca L. Young, Laura Quintana, Harold H. Zakon, Hans A. Hofmann, José Sotelo-Silveira*, Ana Silva*

**EXTENDED METHODS**

Animals

*Gymnotus omarorum* individuals were collected using a fish detector as described elsewhere [^1^](https://paperpile.com/c/4P8b79/gPAH) in Laguna del Sauce (34°51’S, 55°07’W, Department of Maldonado, Uruguay). As sex in *G. omarorum* is not externally apparent (neither morphologically nor electrophysiologically), it was determined before behavioural experiments by gonadal inspection [^2^](https://paperpile.com/c/4P8b79/8CJM). Animals were housed in individual compartments in 500-l outdoor tanks for 10 to 30 days before the behavioural experiments. All environmental variables were kept within the normal range exhibited in the natural habitat in the non-breeding season and fish were fed with *Tubifex tubifex* worms once a week [^3,4^](https://paperpile.com/c/4P8b79/4YMu+yFjP).

The POA sections used for transcriptomic analysis contain, at least partially, the following brain areas: DL (dorsolateral telencephalon), DD (dorsal division of the dorsal forebrain), DM2v (dorsomedial telencephalon, subdivision 2 ventral), DM2d (dorsomedial telencephalon, subdivision 2 dorsal), VP (ventral telencephalon, posterior subdivision), DC (central division of dorsal forebrain), AC (anterior commissure), Vs (ventral telencephalon, supracommissural subdivision), FB (forebrain bundle LFB + MFB), DM (dorsomedial telencephalon, homologous to extended medial amygdala), DPI (lateral subdivision of caudal DP), SC (suprachiasmatic nucleus), PGm (preglomerular nucleus, medial subdivision), PGI preglomerular nucleus, lateral subdivision), TeO (optic tectum), cT: (tectal commissure), TSd (torus semicircularis, dorsal subdivision), Ha (anterior hypothalamus).

Behaviour

Two males with body weight asymmetries between 13.2-24.2% were placed in opposite sides of a 120-L tank (length: 110 cm; width: 80 cm; height: 25 cm) divided in two equally sized compartments by a glass gate (Fig. 2); while 2 extra partitions located in opposite corners prevented any physical or electrical interaction before gate removal (6 h). This experimental tank allowed simultaneous electric and video recordings [^5^](https://paperpile.com/c/4P8b79/9Nza). The electric signals of freely moving fish were detected by two pairs of orthogonal fixed electrodes attached to each tank wall, connected to two high-input impedance amplifiers (FLA-01, Cygnus Technologies Inc.). An infrared-sensitive video camera (SONY CCD-Iris and RoHS CCD Digital Video Camera) focused on the bottom of the tank. Images and electric signals were captured on a video card (EasyCap) and stored in the computer for later analysis. The agonistic encounter started 5 min after the beginning of the dark phase by lifting the 3 partitions and ended 36 h later with light-dark phases of 10:14 h to mimic non-breeding natural photoperiod. In these conditions, space is the only resource that individuals fight for, as we provided symmetric resources and resource values for both contestants: equally-sized plain tanks, same residence time, and the same previous experience as fish were kept in their individual outdoor housing compartments with no physical contact with conspecifics before the experiment [^4^](https://paperpile.com/c/4P8b79/yFjP). The size asymmetry between contenders allowed us to predict the contest outcome [^3^](https://paperpile.com/c/4P8b79/4YMu). We enriched the resource value of the territory by adding one shelter in the middle of the arena. Both video and electric recordings were continuously obtained during the first hour of interaction and every 30 min (in 2 min bin videos) during the next 35 h. The first attack was considered as the beginning of the contest phase, and resolution was achieved when one of the contenders (the subordinate) retreated 3 times without attacking back. After 36 h, we removed and sacrificed both, dominant and subordinate, for transcriptomic studies.

We measured the following locomotor parameters: latency to the first attack, contest duration, attack/retreat rate during contest as the number of attacks/retreats divided by the duration of the contest in min, and LPR attack/retreat rate as the number of attacks/retreats per min recorded in all the 2-min samples taken each 30 min from 60 min to 36 h after resolution (Fig. 3). In addition, during the LPR, the use of the shelter and the occupancy of its surrounding territory was calculated for both contenders to corroborate that the dominant-subordinate status was maintained over time without reversion [^3^](https://paperpile.com/c/4P8b79/4YMu). Shelter occupancy was measured as the percentage of the number of 2-min samples in which each fish was observed inside the shelter divided by the total number of recorded samples. The presence of each fish in the surroundings of the shelter was evaluated by a score calculated as follows: score 5 (inside the shelter), score 3 (inside a circle centred on the middle of the shelter with a diameter twice the shelter length), and score 1 (beyond this circle). The maximum score value was used for each 2 min-sample, and the mean value of all the scores was used as the territory access index for each individual (Fig. 3).

**SUPPLEMENTARY REFERENCES**

1. [Silva, A. C., Quintana, L., Galeano, M. & Errandonea, P. Biogeography and breeding in Gymnotiformes from Uruguay. Environ. Biol. Fishes 66, 329–338 (2003).](http://paperpile.com/b/4P8b79/gPAH)

2. [Jalabert, C., Quintana, L., Pessina, P. & Silva, A. Extra-gonadal steroids modulate non-breeding territorial aggression in weakly electric fish. Horm. Behav. 72, 60–67 (2015).](http://paperpile.com/b/4P8b79/8CJM) 3. [Perrone, R. & Silva, A. C. Status-Dependent Vasotocin Modulation of Dominance and Subordination in the Weakly Electric Fish. Front. Behav. Neurosci. 12, 1 (2018).](http://paperpile.com/b/4P8b79/4YMu)

4. [Batista, G., Zubizarreta, L., Perrone, R. & Silva, A. Non-sex-biased Dominance in a Sexually Monomorphic Electric Fish: Fight Structure and Submissive Electric Signalling. Ethology 118, 398–410 (2012).](http://paperpile.com/b/4P8b79/yFjP)

5. [Silva, A., Perrone, R. & Macadar, O. Environmental, seasonal, and social modulations of basal activity in a weakly electric fish. Physiol. Behav. 90, 525–536 (2007).](http://paperpile.com/b/4P8b79/9Nza)

**SUPPLEMENTARY FIGURES**

**
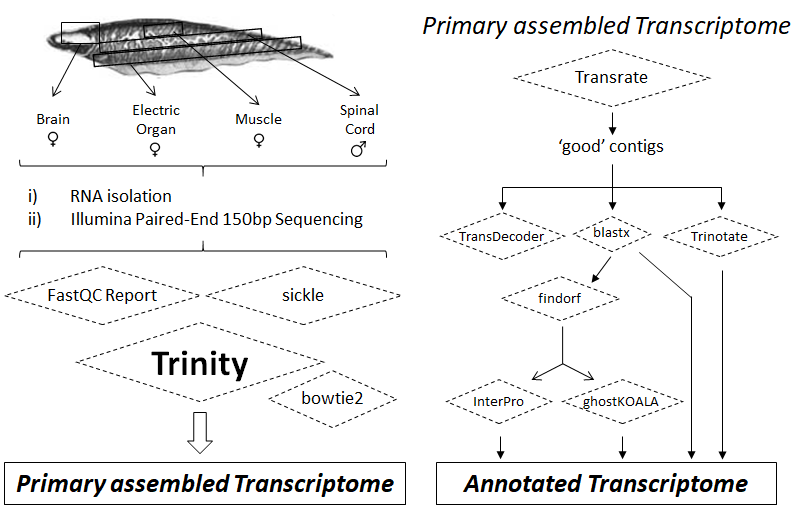
**

**Supplementary Figure S1.** Workflow to produce *Gymnotus omarorum* annotated Transcriptome reference. The workflow in the left panel shows the different samples submitted to Illumina sequencing, from which primary transcriptome was assembled using Trinity. This primary transcriptome was evaluated by Transrate and annotated using several software indicated in the right panel. All information obtained was incorporated to produce an annotated Transcriptome reference of *Gymnotus omarorum* available in fasta (nucleotide and amino acid sequence) and gff format.


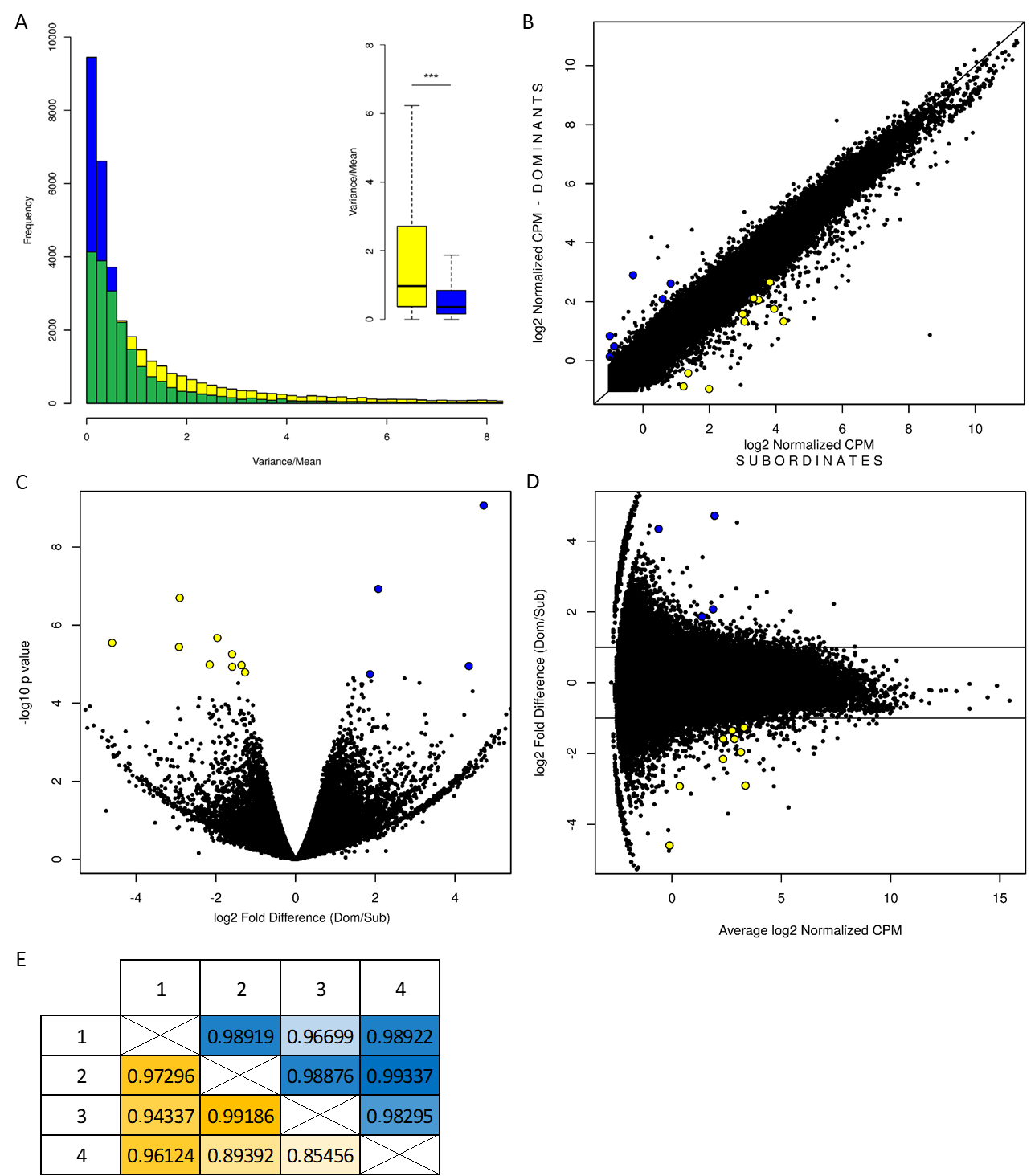


**Supplementary Figure S2.** (**A**) Histogram and boxplot of variance/mean distribution for normalized CPM in Dominants (blue) and Subordinates (yellow). Statistical differences are significant by ANOVA (p value = 8.92E-7). (**B**) Scatter plot showing edgeR normalized CPM in Dominants and Subordinates. (**C**) Volcano plot showing the relationship between fold difference and p values. (**D**) MA plot. (**E**) Inter-replicates Pearson correlation coefficients are shown among samples. Above diagonal are correlations between dominants replicates, while below are between subordinates. In B-D DEGs are shown in colour scheme: in blue overexpressed transcripts in dominants, and in yellow overexpressed in subordinates.


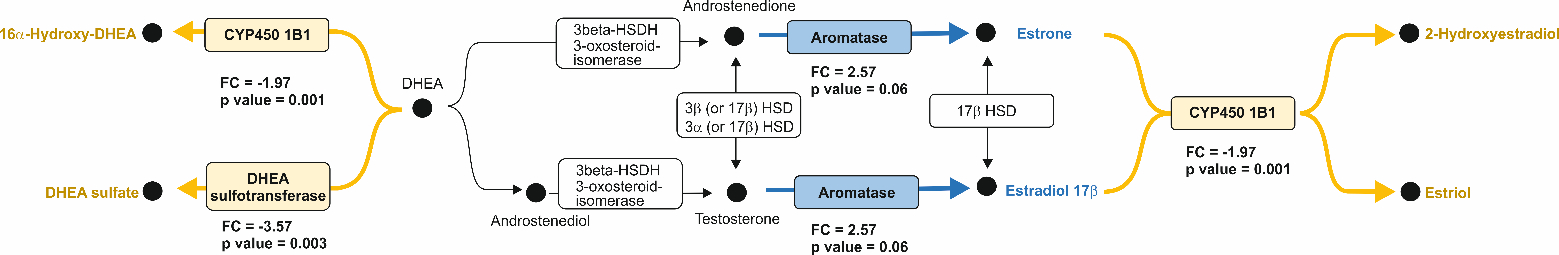


**Supplementary Figure S3.**Dominants and subordinates show differential expression of steroidogenic enzymes. Dominants favoured the conversion of androgens to oestrogens (blue arrows) while subordinates (yellow arrows) promoted the conversion towards non-aromatisable androgens and to steroids with lower estrogen receptor affinity. Dominants showed a marginally higher expression of aromatase transcripts whereas subordinates showed a differential increase in transcripts of enzymes that direct metabolic pathways away from aromatisable androgens and estradiol.

**

**

**Supplementary Figure S4.**Weight does not explain behaviour asymmetries. (**A**) Same-sized fish attack differently according to status: dominants attack more than subordinates regardless of their weight. Also the heaviest dominant displays the same attack rate than one 25% lighter. (**B**) Same as (A) but for territory access scores. In this case, the heaviest dominant fish is not the one that displays the highest score.

**SUPPLEMENTARY TABLES**

**Supplementary Table S1.** Sequencing, trimming and mapping statistics. Table shows raw number of sequenced reads, trimming and mapping statistics for all the replicates of subordinates and dominants fishes.

|  | **Raw Sequences** | **Trimmed PE reads** | **Trimmed SE reads** | **% Trimming** | **Mapped Sequences** | **% Mapping** |
| --- | --- | --- | --- | --- | --- | --- |
| Sub1 | 20,425,236 | 19,774,486 | 302,973 | 98.29 | 9,843,775 | 49.03 |
| Sub2 | 22,915,092 | 22,146,748 | 356,735 | 98.20 | 11,122,494 | 49.43 |
| Sub3 | 29,849,188 | 29,006,512 | 394,509 | 98.50 | 16,210,719 | 55.14 |
| Sub4 | 28,316,792 | 27,460,410 | 400,483 | 98.39 | 12,887,141 | 46.26 |
| Dom1 | 32,382,938 | 31,377,122 | 467,761 | 98.34 | 15,311,431 | 48.08 |
| Dom2 | 27,150,318 | 26,295,700 | 397,174 | 98.32 | 12,807,784 | 47.98 |
| Dom3 | 32,981,968 | 31,911,056 | 495,538 | 98.26 | 15,700,031 | 48.45 |
| Dom4 | 29,882,014 | 28,950,856 | 432,090 | 98.33 | 14,557,772 | 49.54 |

**Supplementary Table S2.** Differentially expressed genes with FDR <0.05.

| **Gen_ID** | **Annotation** | **Average CPM SUB** | **Average CPM DOM** | **log2 FC** | **FDR** |
| --- | --- | --- | --- | --- | --- |
| TRINITY_DN48709_c0_g2_i4 | NA | 0.31 | 6.99 | 4.72 | 2.09E-05 |
| TRINITY_DN49759_c1_g12_i1 | Uromodulin | 0.00 | 1.29 | *NA* | 4.62E-05 |
| TRINITY_DN48116_c1_g1_i2 | Pleckstrin homology-like domain | 1.28 | 5.63 | 2.07 | 1.15E-03 |
| TRINITY_DN187449_c0_g1_i1 | Somatostatin | 18.29 | 2.02 | -2.91 | 1.63E-03 |
| TRINITY_DN47635_c0_g1_i2 | Microfibril-associated glycoprotein | 14.94 | 2.88 | -1.96 | 1.47E-02 |
| TRINITY_DN62267_c0_g1_i1 | Parathyroid hormone | 1.83 | 0.05 | -4.60 | 1.73E-02 |
| TRINITY_DN132072_c0_g1_i1 | Kinetochore-associated protein | 2.07 | 0.25 | -2.93 | 1.96E-02 |
| TRINITY_DN218819_c0_g1_i1 | Hemoglobin subunit beta 2 | 3.46 | 0.02 | -6.19 | 2.22E-02 |
| TRINITY_DN231252_c0_g2_i1 | Homer protein homolog | 10.67 | 3.69 | -1.59 | 2.25E-02 |
| TRINITY_DN8325_c0_g1_i1 | Matrilin 3 | 0.00 | 0.60 | *NA* | 2.25E-02 |
| TRINITY_DN125459_c0_g1_i1 | NA | 0.05 | 0.90 | 4.35 | 3.55E-02 |
| TRINITY_DN17513_c0_g1_i1 | Homeobox protein | 7.80 | 2.01 | -2.16 | 3.55E-02 |
| TRINITY_DN185564_c0_g1_i1 | Fibromodulin | 7.51 | 2.50 | -1.59 | 3.55E-02 |
| TRINITY_DN31315_c0_g1_i1 | Homer protein homolog | 9.52 | 3.83 | -1.36 | 3.55E-02 |
| TRINITY_DN38819_c0_g2_i1 | Prolyl 4-hydroxylase subunit alpha | 13.62 | 5.84 | -1.27 | 4.63E-02 |
| TRINITY_DN222754_c0_g1_i1 | Neuralized 2 | 1.01 | 3.77 | 1.87 | 4.85E-02 |

**Supplementary Table S3.** Metadata of candidate genes overexpressed in dominants. Table shows literature available data of overexpressed genes in dominants zebrafish and their homologous in our data with corresponding values. 

| **Gene** | **FC** | **p val** | **reference** | **sex** | **Gen ID** | **Annotation** | **FC** | **p val** | **bitscore** | **QCOVS** |
| --- | --- | --- | --- | --- | --- | --- | --- | --- | --- | --- |
| AR | 2.61 | 0.014 | Filby | female | TRINITY_DN10652_c0_g1_i1 | Androgen receptor | 1.07 | 0.80 | 0 | 65 |
|  |  |  |  |  | TRINITY_DN41294_c0_g1_i1 | Androgen receptor | 1.52 | 0.03 | 2.00E-170 | 48 |
|  |  |  |  |  | TRINITY_DN41294_c0_g1_i2 | Androgen receptor | 1.59 | 0.03 | 3.00E-173 | 48 |
|  | 1.96 | 0.01 |  | male | TRINITY_DN10652_c0_g1_i1 | Androgen receptor | 1.07 | 0.80 | 0 | 65 |
|  |  |  |  |  | TRINITY_DN41294_c0_g1_i1 | Androgen receptor | 1.52 | 0.03 | 2.00E-170 | 48 |
|  |  |  |  |  | TRINITY_DN41294_c0_g1_i2 | Androgen receptor | 1.59 | 0.03 | 3.00E-173 | 48 |
| AVPR1B | 3.04 | 0.022 | Filby | female | TRINITY_DN38986_c0_g1_i1 | AVT receptor | 1.57 | 0.09 | 0 | 92 |
|  | 1.56 | 0.02 |  | male |  |  |  |  |  |  |
| CRHB | 1.88 | 0.025 | Filby | female | TRINITY_DN126934_c0_g1_i1 | Corticotropin releasing factor | -3.59 | 0.09 | 2.00E-50 | 100 |
| CYP19A1B | 2.95 | 0.001 | Filby | female | TRINITY_DN30466_c0_g2_i1 | Cytochrome P450 (Estrogen synthase) | 2.03 | 0.18 | 0 | 71 |
| ESR1 | 3.02 | 0.005 | Filby | female | TRINITY_DN42642_c0_g2_i1 | Estrogen receptor | -1.38 | 0.13 | 1.00E-59 | 59 |
|  |  |  |  |  | TRINITY_DN42642_c1_g2_i1 | Estrogen receptor | -1.50 | 0.40 | 3.00E-64 | 59 |
| ESR2A | 2.8 | 0.022 | Filby | female | TRINITY_DN42642_c0_g2_i1 | Estrogen receptor | -1.38 | 0.13 | 3.00E-70 | 65 |
|  |  |  |  |  | TRINITY_DN42642_c1_g2_i1 | Estrogen receptor | -1.50 | 0.40 | 5.00E-73 | 65 |
| HDC | 1.88 | 0.003 | Filby | male | TRINITY_DN146268_c0_g1_i1 | Histidine decarboxylase | -1.14 | 0.89 | 4.00E-23 | 10 |
|  | 1.85 | NA | Pavlidis | male | TRINITY_DN220587_c0_g1_i1 | DOPA decarboxylase | -1.94 | 0.01 | 7.00E-163 | 78 |
| HRH2 | 1.96 | 0.029 | Filby | female | TRINITY_DN45249_c0_g1_i1 | Histamine H2 receptor | 1.16 | 0.55 | 1.00E-47 | 45 |
| HTR1AA | 1.59 | 0.027 | Filby | male | TRINITY_DN123508_c0_g1_i1 | Hydroxytryptamine receptor 1B | -1.26 | 0.40 | 3.00E-88 | 99 |
|  |  |  |  |  | TRINITY_DN171152_c0_g1_i1 | Hydroxytryptamine receptor 1A | -1.39 | 0.45 | 7.00E-79 | 38 |
|  |  |  |  |  | TRINITY_DN86812_c0_g1_i1 | Hydroxytryptamine receptor 1F | -1.07 | 0.83 | 2.00E-70 | 87 |
| MAO | 3.52 | 0.014 | Filby | female | TRINITY_DN48373_c0_g4_i2 | Monoamine oxidase | -1.30 | 0.15 | 0 | 99 |
|  |  |  |  |  | TRINITY_DN48373_c0_g4_i3 | Monoamine oxidase | -1.36 | 0.09 | 0 | 97 |
| NOS1 | 2.51 | 0.022 | Filby | female | TRINITY_DN46474_c2_g3_i1 | NADPH | -1.48 | 0.04 | 1.00E-58 | 45 |
| NPY | 4.37 | 0.002 | Filby | female | TRINITY_DN239730_c0_g1_i1 | Peptide YY | 4.68 | 0.01 | 6.00E-28 | 100 |
| NR3C1 | 2.02 | 0.04 | Filby | female | TRINITY_DN31664_c0_g1_i2 | Mineralocorticoid receptor | 1.22 | 0.40 | 1.00E-118 | 54 |
|  |  |  |  |  | TRINITY_DN41294_c0_g1_i1 | Androgen receptor | 1.52 | 0.03 | 2.00E-112 | 48 |
|  |  |  |  |  | TRINITY_DN41294_c0_g1_i2 | Androgen receptor | 1.59 | 0.03 | 1.00E-114 | 48 |
|  |  |  |  |  | TRINITY_DN48993_c1_g2_i1 | Glucocorticoid receptor | 1.15 | 0.44 | 9.00E-156 | 34 |
|  |  |  |  |  | TRINITY_DN48993_c4_g2_i2 | Glucocorticoid receptor | 1.09 | 0.64 | 2.00E-132 | 55 |
| NR3C2 | 1.5 | NA | Pavlidis | male | TRINITY_DN31664_c0_g1_i1 | Mineralocorticoid receptor | 1.10 | 0.66 | 0 | 80 |
|  |  |  |  |  | TRINITY_DN31664_c0_g1_i2 | Mineralocorticoid receptor | 1.22 | 0.40 | 0 | 92 |
| SLC6A3 | 2.52 | 0.02 | Filby | female | TRINITY_DN46481_c0_g1_i1 | Sodium and chloride dependent glycine transporter 2 | 1.25 | 0.82 | 4.00E-136 | 87 |
|  |  |  |  |  | TRINITY_DN46481_c0_g1_i2 | Sodium and chloride dependent glycine transporter 2 | 1.85 | 0.30 | 3.00E-136 | 87 |
| SLC6A4A | 3.44 | 0.001 | Filby | female | TRINITY_DN46481_c0_g1_i1 | Sodium and chloride dependent glycine transporter 2 | 1.25 | 0.82 | 4.00E-141 | 93 |
|  |  |  |  |  | TRINITY_DN46481_c0_g1_i2 | Sodium and chloride dependent glycine transporter 2 | 1.85 | 0.30 | 6.00E-141 | 93 |
| SST1.1 | 1.77 | 0.015 | Filby | female | TRINITY_DN159799_c0_g1_i1 | Somatostatin | 2.03 | 0.09 | 7.00E-44 | 100 |
| SSTR1A | 1.67 | 0.019 | Filby | male | TRINITY_DN119797_c0_g1_i1 | Somatostatin receptor 2 | 1.23 | 0.43 | 6.00E-97 | 97 |
|  |  |  |  |  | TRINITY_DN30512_c0_g1_i1 | Somatostatin receptor 1 | -1.04 | 0.83 | 0 | 100 |
| TH | 2.1 | 0.038 | Filby | female | TRINITY_DN112261_c0_g1_i1 | Tyrosine hydroxylase | -1.05 | 0.84 | 6.00E-107 | 44 |
|  | 1.94 | 0.021 |  | male |  |  |  |  |  |  |
| TPH1B | 2.98 | 0.017 | Filby | male | TRINITY_DN217471_c0_g1_i1 | Tryptophan hydroxylase | -2.22 | 0.03 | 4.00E-146 | 47 |
|  |  |  |  |  | TRINITY_DN31736_c0_g1_i1 | Tryptophan hydroxylase | -3.97 | 0.03 | 2.00E-116 | 56 |
| TPH2 | 2.96 | 0.005 | Filby | female | TRINITY_DN217471_c0_g1_i1 | Tryptophan hydroxylase | -2.22 | 0.03 | 3.00E-138 | 45 |
|  |  |  |  |  | TRINITY_DN31736_c0_g1_i1 | Tryptophan hydroxylase | -3.97 | 0.03 | 0 | 60 |

**Supplementary Table S4.** Pearson correlation values, with associated p value, between transcriptomic signature genes (Fig. 5) expression values and behaviour traits evaluated.

|  | Latency (dom) | Duration (sub) | Duration (dom) | Attacks/min (all) | Retreats/min (sub) | Shelter Occupancy (dom) | Territory Score (all) |
| --- | --- | --- | --- | --- | --- | --- | --- |
| Serotonin Receptor 2A | 0.996 (0.000) |  |  | -0.736 (0.029) |  | 0.967 (0.006) |  |
| NPY | -0.924 (0.027) |  |  |  |  |  |  |
| Dopamine Receptor D2 | 0.975 (0.003) |  |  |  |  | 0.913 (0.034) |  |
| Galanin |  |  |  |  | 1.000 (0.000) |  |  |
| GABA receptor subunit β3 |  |  | 0.900 (0.043) |  |  | -0.956 (0.010) |  |
| Sulfotransferase 2B |  |  |  |  | -0.958 (0.009) |  |  |
| CD59 antigen |  |  | -0.907 (0.038) |  |  |  |  |
| Dynamin-1 | -0.993 (0.000) |  |  |  |  | -0.973 (0.004) |  |
| Glutamate Receptor NMDA 1 | -0.908 (0.037) |  |  |  |  | -0.970 (0.005) |  |
| GREB1 |  | 0.940 (0.018) |  | 0.749 (0.024) |  | 0.893 (0.049) | 0.906 (0.001) |
| Somatostatin |  |  |  | -0.728 (0.031) |  |  |  |
